# A phase II study of UFT with leucovorin administered as a twice daily schedule in the treatment of patients with metastatic colorectal cancer

**DOI:** 10.1038/sj.bjc.6604541

**Published:** 2008-08-12

**Authors:** P M Hoff, S Kopetz, M B Thomas, A Langleben, D Rinaldi, L Anthony, R A Wolff, Y Lassere, J L Abbruzzese

**Affiliations:** 1 Department of Gastrointestinal Medical Oncology, The University of Texas M. D. Anderson Cancer Center, Houston, Texas 77030, USA; 2 Discipline of Gastrointestinal Surgery, University of Sao Paulo, Sao Paulo, Brazil; 3 Hospital Sirio Libanes, Sao Paulo, Brazil; 4 Department of Oncology, Royal Victoria Hospital, Montreal, Quebec, Canada; 5 Louisiana Oncology Associates, Lafayette, Louisiana 70506, USA; 6 Louisiana State University Health Sciences Center, New Orleans, Louisiana 70112, USA

**Keywords:** UFT, dosing, twice daily, thrice daily, metastatic colorectal cancer

## Abstract

Prolonged infusions have been shown to be safer and potentially more effective than bolus regimens of 5-fluorouracil (5-FU) as treatment for metastatic colorectal cancer (mCRC). However, infusional 5-FU requires central venous access and costly infusion pumps. Oral fluoropyrimidines enable longer exposures to 5-FU with increased convenience. Tegafur–uracil (UFT) with leucovorin (LV) given thrice daily has improved safety plus comparable survival and response rates to bolus 5-FU/LV. We conducted a phase II clinical study in 98 patients with mCRC to evaluate if UFT with LV given twice daily provided comparable time to progression (TTP), efficacy and tolerability to that reported for thrice daily in two phase III clinical studies. Secondary objectives included overall response rate (ORR) and overall survival (OS). Median TTP was 3.8 months, when compared with 3.5 months for thrice daily. The ORR (11%) and median OS (12.8 months) with twice daily administration were similar to that of thrice daily administration (12% and 12.4 months). The incidence of grade 3/4 treatment-related diarrhoea was 30% on the twice daily and 21% on the thrice daily schedule. These results suggest that twice daily administration has similar efficacy and tolerability to thrice daily administration and is an acceptable alternative for patients who would benefit from UFT with LV therapy.

Despite the recent advances in treatment, colorectal cancer (CRC) remains the second leading cause of cancer death in the United States (Jemal *et al*, 2007). Most CRCs are adenocarcinomas, and the primary method of treatment is surgery, which can result in cure, depending on the stage of the disease at the time of diagnosis. Metastatic CRC (mCRC) is usually treated with 5-fluorouracil (5-FU)-based chemotherapy, either 5-FU alone or in combination with modulators and other cytotoxic agents, such as irinotecan and oxaliplatin (Kopetz and Hoff, 2005).

A meta-analysis of randomised clinical studies comparing 5-FU regimens for the treatment of mCRC showed that continuous infusion was superior to bolus administration in terms of tumour response (22 *vs* 14%) and median survival (12.1 *vs* 11.3 months) (Meta-Analysis Group in Cancer, 1998). After entering the circulation, most of the infused 5-FU is rapidly converted to inactive metabolites by dihydropyrimidine dehydrogenase (DPD) (Diasio, 2001). The remainder is converted to fluorouridine monophosphate, which is subsequently converted to fluorouridine diphosphate and then to either fluorouridine triphosphate (FUTP) or fluorodeoxyuridine monophosphate (FdUMP) (Schilsky, 1992). The mechanisms of 5-FU cytotoxicity include the inhibition of DNA synthesis through the inhibition of thymidylate synthase (TS) by FdUMP (Peters *et al*, 1989) and the inhibition of RNA synthesis when FUTP is incorporated into RNA (Daher *et al*, 1990; Hoff *et al*, 1999a). The side effects of bolus 5-FU and the inconvenience of prolonged infusions have led investigators to search for delivery alternatives for 5-FU, including such oral prodrugs as tegafur, tegafur–uracil (UFT) and capecitabine (Hoff *et al*, 1999a).

Tegafur is a fluorinated pyrimidine first synthesised by Hiller *et al* (1967). After showing significant neurotoxicity when administered intravenously (Buroker *et al*, 1979), it was developed in Japan as an oral formulation. It can be orally administered because it is absorbed as an intact molecule and is not metabolised by DPD in the gastrointestinal tract. Once tegafur is metabolised to 5-FU, mostly in the liver, it enters the same pathway as intravenous (i.v.) 5-FU and has the same cytotoxic mechanism of action (Hoff *et al*, 1999a).

Tegafur–uracil, a combination of tegafur and uracil in a 1 : 4 molar ratio, is a further development of the concept. Uracil is naturally metabolised by DPD and competes with 5-FU for the enzyme when the two are administered together, resulting in a significantly prolonged half-life for 5-FU, mimicking continuous infusion (Hoff *et al*, 1998). Leucovorin (LV), also known as folinic acid, is a tetrahydrofolic acid derivative that can enhance the therapeutic effects of fluoropyrimidines such as 5-FU by stabilising the FdUMP/TS complex and enhancing the inhibition of DNA synthesis (Tsai *et al*, 1990; Ardalan *et al*, 1998). It is combined with UFT to further potentiate the effect of 5-FU on tumour cells (Hoff *et al*, 1999b).

Two phase III studies in previously untreated patients with mCRC compared a regimen of UFT 300 mg m^–2^ day^–1^ with LV 75–90 mg thrice daily for 28 days and repeated every 35 days against the standard Mayo Clinic regimen of intravenous 5-FU 425 plus LV 20 mg m^–2^ day^–1^ for 5 days repeated every 28 (Douillard *et al*, 2002) or 35 days (Carmichael *et al*, 2002). Patients receiving UFT with LV experienced less diarrhoea and mucositis than those who received 5-FU/LV and showed similar response rates, time to progression (TTP) and median overall survival (OS).

The daily dose of UFT with LV is usually divided into three daily doses (Pazdur *et al*, 1998). Despite this requirement for thrice daily administration, UFT was preferred to bolus 5-FU by the majority of patients, mainly due to convenience, in a quality-of-life (QoL) study in the treatment of mCRC (Sizer *et al*, 2006). An UFT twice daily dosing schedule, which would potentially be more convenient and have a positive effect on patients’ QoL, has been shown to be as well tolerated as thrice daily dosing (Etienne-Grimaldi *et al*, 2007). In this phase II pharmacokinetic study, 21 patients with mCRC were randomised to receive UFT 300 mg m^–2^ day^–1^ plus LV 90 mg m^2^ day^–1^ on days 1–28 of a 35-day cycle either twice or thrice daily for the first cycle. Patients were then crossed over to the other dose schedule for the second cycle. Twice daily dosing resulted in a twofold increase in the fluorouracil and uracil AUC values but was as well tolerated as thrice daily dosing, suggesting that this more convenient schedule may improve the UFT therapeutic index.

To evaluate the feasibility of a more convenient twice daily schedule, we conducted a phase II study of UFT with LV administered twice daily for 28 days, repeated every 35 days, for the treatment of patients with mCRC. The total daily UFT dose (300 mg m^–2^) was the same as that investigated in two phase III studies with thrice daily dosing (Carmichael *et al*, 2002; Douillard *et al*, 2002).

## Patients and methods

### Patients

This was a non-randomised, multicentre, open-label phase II study. The eligibility criteria included age ⩾18 years, histologically confirmed and measurable metastatic colorectal adenocarcinoma, Eastern Cooperative Oncology Group (ECOG) performance status ⩽2, life expectancy >12 weeks, adequate haematological, renal and hepatic function and no prior treatment for metastatic disease. Patients who had received prior adjuvant treatment must have completed their adjuvant treatment at least 6 months before study enrolment. All women had to have a negative pre-study serum or urine pregnancy test, unless they were postmenopausal or had been surgically sterilised. Any women of childbearing potential had to practise adequate contraception during the study. All manifestations of toxicity from previous therapy must have returned to baseline levels.

Ineligibility criteria included prior treatment for metastatic disease with the exception of radiotherapy to treat local symptomatic lesions, concomitant use of another investigational drug, a history of brain metastases or carcinomatous meningitis, prior exposure to oral fluoropyrimidines and a history of other cancers with the exception of basal cell skin cancers, carcinoma *in situ* of the cervix or curatively treated cancers that had not recurred for more than 5 years. Patients with an active serious infection, an underlying medical condition that would impair their ability to receive the protocol treatment, dementia or significantly altered mental status that would prohibit the understanding and provision of informed consent and patients who were breast feeding were also excluded. All participating institutions obtained institutional review board (IRB) approval. All patients were informed of the investigational nature of the study and signed an IRB-approved, informed-consent document.

### Treatment

Tegafur–uracil was administered as 100 mg capsules and LV was administered as 15 mg tablets supplied by Bristol-Myers Squibb (Wallingford, CT, USA). Patients received UFT 300 mg m^–2^ day^–1^ with LV 30 mg per dose, twice a day divided into two daily doses administered every 12 h for 28 days. The total daily UFT dose was rounded up or down to the nearest 100 mg. Patients were instructed not to consume any food for 1 h before and 1 h after the study medication was taken.

The dose of UFT for each cycle was reduced by 50 mg m^–2^ day^–1^ if patients had adverse events of grade 2 or higher according to the National Cancer Institute Common Toxicity Criteria scale, version 2.0 (Table 1). Reductions were based on the occurrence of a grade 2 or higher adverse event in two consecutive cycles. If the adverse event was grade 3, UFT was withheld until the event returned to baseline levels. No changes were made to the LV dose; however, if UFT was withheld, LV was also withheld. Treatment could be delayed for up to 2 weeks to allow for recovery from adverse events. If doses were withheld because of adverse events, the days that therapy was omitted were still counted as part of the 28-day treatment cycle. If UFT was withheld for adverse events during a treatment period and the adverse events resolved, the patient resumed treatment at the same dose level that was used before the dose was withheld.

### Patient evaluation

All patients were evaluated with a complete medical history and physical examination, including performance status, radiographic imaging and laboratory tests. Patients were contacted weekly by telephone to elicit information regarding adverse events and compliance. Adverse events were assessed directly during the scheduled office visit each cycle. Patients completed daily diaries in which they recorded the time each dose of medication was taken and all perceived adverse events.

Tumour response was evaluated every two cycles and continued until disease progression. A complete response (CR) was defined as the complete disappearance of all tumour lesions and normalisation of tumour markers. A partial response (PR) was defined as a decrease of >50% in the sum of the products of the two largest perpendicular diameters of all measurable lesions, with no lesions progressing and no new lesions appearing. Stable disease (SD) was defined as a lack of response, as defined above, in the absence of any progressive disease (PD), which was defined as a >25% increase in the size of any measurable or evaluable lesion, the appearance of any new lesions or the occurrence of malignant pleural effusion or ascites. Death secondary to malignant disease was documented as PD. Objective responses were documented with a repeat measurement performed as close to 4 weeks from the original assessment as feasible.

Treatment continued until disease progression, unacceptable toxicity or withdrawal of consent. For patients who remained progression free for 1 year, continuation of therapy was left to the investigator's discretion. Time to progression was calculated for all patients from the date of beginning the study until the date that PD or death was first reported. Data from patients whose disease did not progress were censored at the last date they were known to be alive. Patients who died of disease and for whom a date of progression was not available were considered to have had progressed on the day of death. For all patients, survival was calculated from the date of beginning the study to the date of death. Data from patients who did not die were censored at the date they were last known to be alive.

### Statistical methods

The primary end point of this study was to evaluate the TTP and the proportion of patients who remained progression free at 6 months. Planned secondary end points included ORR, OS and tolerability. On the basis of the results of the phase III UFT thrice daily studies (Carmichael *et al*, 2002; Douillard *et al*, 2002), the proportion of patients who would be progression free at 6 months was estimated to be 30%. Assuming an exponential distribution of TTP, this equates to a median TTP of 3.45 months and a per month hazard rate of 0.201. The total sample size planned was 90 eligible patients, which allowed for estimating the true hazard rate to within 0.082 in that the 95% confidence interval (CI) would be 0.162–0.244 for an observed hazard rate of 0.201. In this design, the twice daily UFT schedule would be considered non-inferior to the thrice daily schedule if the TTP was >2.8 months (corresponding to the upper confidence limit of 0.244 for the per month hazard rate) with a one-sided test for non-inferiority at the 5% significance level. The maximum likelihood estimate was used to calculate median TTP, although, as a secondary analysis, the Kaplan–Meier estimate was also used to show the empirical distributions of TTP and OS. All eligible patients were included in these analyses.

## Results

A total of 98 patients were enrolled in this study and treated between September 1999 and December 2001. Patient demographics are listed in Table 2. All patients received at least one dose of UFT with LV and were evaluable for safety. Seven patients had a protocol violation: one patient received adjuvant tegafur, one patient had progressed within 6 months of receiving adjuvant treatment, two patients had lesions smaller than the required 1.5 cm and three patients had raised baseline levels of bilirubin or liver enzyme levels. However, 97 patients were included in the efficacy analysis. A total of 394 cycles were administered, with a mean of four cycles per patient (range: 1–17). Seventy-one patients (73%) discontinued treatment because of PD, six patients (6%) refused further treatment, five patients (5%) were removed from the study by the investigator, six patients (6%) were removed from the study because of adverse events, six patients (6%) died during the study, three patients (3%) were removed from the study because of concurrent illness and one patient (1%) was removed because he had a liver resection.

All patients experienced at least one adverse event, the most common being asthenia (75% of patients), diarrhoea (66%) and nausea (57%). Forty-three patients had at least one grade 3/4 treatment-related adverse event. The most frequent grade 3/4 adverse events considered to be treatment related were diarrhoea (30% of patients), asthenia (12%), dehydration (9%), nausea (5%) and vomiting (4%). Four patients (4%) had grade 3 anaemia and seven (7%) had grade 3/4 hyperbilirubinaemia (six grade 3 and one grade 4). Seven patients (7%) were removed from the study because of adverse events (two with severe diarrhoea, two with severe dehydration, one with severe fatigue and abdominal pain, one with hyperbilirubinaemia and one with severe nausea and vomiting). A total of 13 patients died either during the study or within 30 days of last receiving study medication. Two deaths were considered to be treatment related. The first was a 79-year-old patient with severe diarrhoea followed by renal failure and the second was an 82-year-old patient with severe diarrhoea followed by sepsis. The presumed cause of death for all 13 cases is listed in Table 3.

Table 4 summarises the tumour response for all patients included in the study. Two patients (2%) had a CR and nine patients (9%) had a PR (95% CI: 4.7–16.8). The ORR was 11% (95% CI: 6.1–19.2%). Thirty-nine patients (40%) had SD (95% CI: 30.3–50.1%) and 31 patients (32%) had PD (95% CI: 22.9–41.7%). Seventeen patients (17%) were not evaluable for tumour response because they did not complete two cycles of study therapy or had no clear evidence of clinical progression; they were, however, included in the denominator for calculating response rate.

The median TTP was 3.8 months (95% CI: 3.11–4.65) and the percentage of patients’ progression free at 6 months was 33% based on an exponential distribution. On the basis of the secondary Kaplan–Meier analysis, the median TTP was 4.5 months and the proportion of patients progression-free at 6 months was 29%. Using the Cox proportional hazards regression model, none of the following variables was found to be a significant predictor of TTP: patient age, performance status, primary disease location, prior adjuvant therapy and the interval between adjuvant therapy and the diagnosis of metastatic disease. The median OS based on the Kaplan–Meier methodology was 12.8 months (95% CI: 9.6–15.8).

## Discussion

The rationale for this study was to evaluate whether the twice daily schedule for the administration of UFT provided similar efficacy and tolerability compared with the thrice daily schedule. We estimated that the proportion of patients who would be progression free at 6 months would be 30%, which would equate to a median TTP of 3.45 months. On the basis of the 97 eligible patients in our study, 33% were progression-free at 6 months and the median TTP was 3.8 months. The Kaplan–Meier estimator was also used to show the empirical distribution of TTP as a secondary analysis, showing that 29% of the patients were progression free at 6 months and the median TTP was approximately 4.5 months. The median TTP for thrice daily UFT in the two phase III studies was 3.4 and 3.5 months, the ORR was 11 and 12% and median OS was 12.2 and 12.4 months (Carmichael *et al*, 2002; Douillard *et al*, 2002). The results of our study indicate that UFT with LV given as a twice daily schedule for the treatment of patients with mCRC has similar antitumour activity to that seen with the thrice daily schedule in two phase III studies (Carmichael *et al*, 2002; Douillard *et al*, 2002). The median TTP of 3.8 months in our study is non-inferior to 3.4 and 3.5 months reported for the thrice daily schedules in two phase III studies (Carmichael *et al*, 2002; Douillard *et al*, 2002). In addition, the ORR and median OS for the twice daily schedule were similar to those reported for the thrice daily schedule.

As expected, grade 3/4 haematological events were uncommon in our study and were limited to four patients (4%) with grade 3 anaemia. The most frequent grade 3/4 treatment-related non-haematological adverse events were diarrhoea (30%) and asthenia (12%). The tolerability profile of the twice daily schedule appears to be similar to that reported for the thrice daily schedule in the phase III clinical studies and the most common adverse events with both schedules were gastrointestinal. The incidence of grade 3/4 treatment-related diarrhoea and nausea/vomiting was 21 and 13%, respectively, with the thrice daily schedule (Carmichael *et al*, 2002; Douillard *et al*, 2002) compared with 30 and 9%, respectively, with the twice daily schedule. No cases of hand-foot syndrome were reported in one study (Carmichael *et al*, 2002), and in the other study (Douillard *et al*, 2002), only eight patients (2%) had mild symptoms and no patients had severe hand-foot syndrome. There was no significant hand-foot syndrome in our study.

Although two patients (2%) died from severe diarrhoea in our study, the overall treatment-related mortality for this schedule is consistent with that seen with the use of other chemotherapy regimens in mCRC (Rothenberg *et al*, 2001). A polymorphism in CYP2A6, a cytochrome P450 enzyme reported to metabolise tegafur to 5-FU, has been demonstrated in a recent report of one patient treated with UFT who developed severe diarrhoea and fatal sepsis (Bosch *et al*, 2007). It was hypothesised in this report that altered metabolism due to this polymorphism resulted in increased toxicity.

The efficacy of twice daily UFT with LV also appears to be similar to that reported for twice daily capecitabine in two phase III studies of first-line treatment for mCRC, with ORRs of 19 and 26%, median TTPs of 4.3 and 5.2 months, and median OS of 12.5 and 13.2 months (Hoff *et al*, 2001; Van Cutsem *et al*, 2001).

In conclusion, our results suggest that the twice daily schedule of UFT with LV provides similar efficacy and tolerability to that of the thrice daily schedule for the treatment of patients with mCRC and is a reasonable alternative for patients who would benefit from UFT with LV therapy.

## Figures and Tables

**Table 1:** UFT dose levels

**Dose level**	**UFT (mg m^–2^ day^–1^)**
0	300
–1	250
–2	200

**Table 2:** Baseline patient demographics

**Characteristic**	**No. of patients (*n*=98)**	**%**
Median age, years (range)	64 (38–93)	
		
*Sex*		
Male	58	59
Female	40	41
		
*Race*		
White	73	75
Black	12	12
Hispanic	7	7
Other	6	6
		
*ECOG performance status*		
0	34	35
1	49	50
2	15	15
		
*Prior therapy*		
Chemotherapy	24	25
Immunotherapy	1	1
Radiotherapy	13	13
		
*Primary tumour site*		
Colon	81	83
Rectum	17	17
		
*Metastatic site*		
Liver	81	83
Lung	29	30
Lymph nodes	21	21
Other	35	36

**Table 3:** Deaths during treatment and within 30 days after the last dose of UFT with LV

**Cause of death**	**No. of patients**
Progressive disease	7
Diarrhoea^a^	2
Chronic obstructive pulmonary disease	1
Myocardial infarction	1
Pneumonia (non-neutropenic patient)	1
Cerebrovascular accident	1

a One case leading to renal failure and one leading to sepsis. These two deaths were considered to be treatment related.

**Table 4:** Best response to therapy

**Response**	**No. of patients (*n*=98)**	**%**
*Overall response rate*	11	11
Complete response	2	2
Partial response	9	9
Stable disease	39	40
Progressive disease	31	32
Not evaluable	17	17
